# Gene Therapy for Liver Cancers: Current Status from Basic to Clinics

**DOI:** 10.3390/cancers11121865

**Published:** 2019-11-25

**Authors:** Kenya Kamimura, Takeshi Yokoo, Hiroyuki Abe, Shuji Terai

**Affiliations:** Division of Gastroenterology and Hepatology, Graduate School of Medical and Dental Sciences, Niigata University, 1-757, Aasahimachi-Dori, Chuo-Ku, Niigata 9518510, Japan; t-yokoo@med.niigata-u.ac.jp (T.Y.); hiroyukiabe@med.niigata-u.ac.jp (H.A.); terais@med.niigata-u.ac.jp (S.T.)

**Keywords:** gene therapy, liver, cancer, hepatocellular carcinoma, metastatic liver tumors

## Abstract

The liver is a key organ for metabolism, protein synthesis, detoxification, and endocrine function, and among liver diseases, including hepatitis, cirrhosis, malignant tumors, and congenital disease, liver cancer is one of the leading causes of cancer-related deaths worldwide. Conventional therapeutic options such as embolization and chemotherapy are not effective against advanced-stage liver cancer; therefore, continuous efforts focus on the development of novel therapeutic options, including molecular targeted agents and gene therapy. In this review, we will summarize the progress toward the development of gene therapies for liver cancer, with an emphasis on recent clinical trials and preclinical studies.

## 1. Introduction

The liver is the largest organ in the body and has vital functions in protein synthesis, metabolism, and detoxification that play key roles in maintaining homeostasis. There are a number of serious liver diseases including hepatitis, fibrosis, genetic diseases, metabolic diseases, and liver cancer, which is one of the leading causes of cancer-related deaths worldwide [1,2,3].

### 1.1. Liver Cancers

The primary liver cancer is mainly hepatocellular carcinoma (HCC) [2], and its etiology includes viral hepatitis, alcohol addiction, and metabolic diseases [4]. Therefore, HCC can occur in diseased liver and involves various molecular pathways [5]. Other primary liver cancers include cholangiocarcinoma, fibrolamellar carcinoma, hepatoblastoma, angiosarcoma, and other mesenchymal cancers of the liver [2,6]. Secondary liver cancers include metastatic tumors from the breast, lung, pancreas, and colorectal cancers [6].

#### 1.1.1. Hepatocellular Carcinoma

In HCC, which consists of more than 90% of primary liver cancers [2,3], the consideration of the remaining hepatic function is essential in the determination of the therapeutic options [7,8,9]. In other words, HCC patients with poor hepatic reserve function have imitated therapeutic options [3]. HCC is a highly heterogeneous cancer, which has recently been demonstrated by high-throughput sequencing and gene expression profiling, at both the molecular and histological level [10]. Although conventional therapeutic options of surgery, ablation, chemoembolization, systemic chemotherapy, and molecularly targeted agents are partly effective for HCC, they are not sufficient for advanced-stage HCC in terms of its efficacy. The effectiveness of chemotherapy in HCC is restricted by chemo-resistance and systemic side effects. To improve the efficacy and safety of chemotherapeutics in HCC management, targeted carriers such as nanoparticles have been tested for efficacy in basic research, but are not yet sufficient to take to clinics [11]. Recent development in the field of molecular targeted agent (MTA) has shed light on chemotherapy for HCC [12] with the consideration of the molecular expression differences in the tumor. However, these MTAs also have limitations owing to the heterogeneity of HCC, and signaling pathway-specific inhibitors, such as those inhibiting fibroblast growth factor (FGF) 19-FGFR4 signaling pathways, are used in clinical trials [13]. Immune checkpoint inhibitors have also been tested [14], but have shown low efficacy in HCC as a current strategy, and further modification of the immune environment is essential [15,16,17]. Overall, HCC is characterized by heterogeneity [18,19,20,21], high risk of recurrence, and drug resistance. Therefore, with the large number of cases worldwide, new approaches are required for early diagnosis, real-time monitoring, molecular-based diagnosis, and definition of therapeutic targets and effective treatment.

#### 1.1.2. Metastatic Liver Cancer

Metastatic liver tumors can be diagnosed more often than primary liver cancers. Therefore, the selective therapeutic options for the tumor in the liver have been considered in combination with the therapy for the primary lesions including the breast, lung, pancreas, and colorectal cancers [6]. In addition, currently, the genomic information in the tumor tissues can be easily obtained using next generation sequencing.

On the basis of these significant developments and advances in the techniques of molecular biology [5,22], innovative basic research and clinical trials, focusing on the development of gene therapy for liver cancers, are increasing [23,24]; together, these have the potential to offer long therapeutic benefit and overcome the issue of heterogeneity.

### 1.2. Gene Therapy

In a disease where novel therapeutic innovations are sorely needed, combining novel therapies of gene therapy approaches with the currently available cytotoxic chemotherapeutic drugs and radiation therapy [25,26,27] also provides hope for better outcomes in patients with advanced-stage liver cancers, including primary and secondary liver cancers.

In this review, we will summarize the progress toward the development of gene therapies for liver cancers including both primary and secondary tumors, with an emphasis on recent clinical trials and preclinical studies including the gene delivery procedures. In addition, clinical trials focusing on gene expression-based diagnosis and the decision of therapeutic options to apply personalized medicine are summarized based on the information available [28]. Therefore, this review summarizes the current landscape of ongoing and completed clinical studies on liver cancers, provides a review of the basic research, and will help both the physicians and researchers to conduct clinical trials and develop novel gene therapy.

## 2. Gene Therapy for the Liver Cancers

### 2.1. Liver-Directed Gene Therapy

Because of its sophisticated and important function in maintaining homeostasis, various diseases originate in the liver. These diseases include genetic disorders such as hemochromatosis, hemophilia A and B, alpha 1 antitrypsin deficiency, Wilson’s disease, Crigler–Najjar syndrome type I, ornithin transcarbamylase deficiency, type IIa familial hypercholesterolemia, and afibrogenemia. Therefore, basic studies focusing on the genetics-based diagnosis of these diseases; molecular biology studies on the mechanisms of these diseases; and the development of genetics-based therapeutic options, “gene therapy”, for these liver diseases have been extensively studied. Gene therapy has evolved as a potent means of treatment for pathogenic gene defects to achieve normal status. The strategies used to treat disease by gene therapy include gene replacement; gene repair; gene augmentation; gene silencing; vaccination; and, currently, gene editing technology [29,30,31,32].

In addition, the liver has unique anatomical characteristics with three vascular systems of the portal vein, hepatic artery, and hepatic veins and bile ducts, and consists of various types of cells including hepatocytes, endothelial cells in the sinusoids, Kupffer cells, dendritic cells, stellate cells (Ito cells), macrophages, natural killer cells (pit cells), and cholangiocytes. The perisinusoidal space between the endothelial cells in the sinusoid and the hepatocytes is called the “Space of Disse”, and the endothelial cells have fenestrae of about 100 nm in diameter on their surface, which brings blood substances or particles into the space and in contact with the hepatocytes [1]. Therefore, the primary barrier for nucleic acid delivery to the hepatocytes is the plasma membrane and the endothelium, in cases where the molecules are larger than 100 nm and the gene delivery methods used to reach the liver through these anatomical routes and cells have been studied previously [25,27]. The challenges for successful gene therapy for liver cancer are to deliver gene/nucleic acids and cells to the cancer cells without causing irreversible tissue damages. The current gene delivery methods used in liver diseases are summarized in Table 1.

### 2.2. Target Genes for HCC Gene Therapy

With increased knowledge of the molecular-based analyses for HCC [33,34], various basic studies are ongoing to develop novel gene therapy for HCC. These studies include epigenetic changes [35], integration in the genome [36], protein expression [37], endothelial growth factor receptor (EGFR) signaling pathway [38], EGFR/ Hypoxia Inducible Factor-1α/vascular EGF (VEGF) pathways under hypoxic conditions [39,40,41], target gene discovery through copy number alteration [42], and genomic profiling of rare liver cancer [43]. Among them, copy number alterations are one of the most common alterations of cancer cells that result in gain or loss of function of oncogene and tumor suppressor genes; therefore, these can be a target of gene therapy as well as genetic markers [18].

Progress has been made in overcoming the issue of the heterogeneity of liver cancer by utilizing genomic information, two-dimentional culture systems, and patient-derived primary cancer cells [44,45]. The exome and whole cancer genome analyses in human HCC frequently identify mutated somatic genes, including *p53, CTNNB1, AXIN1, ARID1A, ARID2, MLL, MLL2, MLL3, MLL4, IRF2, ATM, CDKN2A,* and *FGF19* [42]. Potential therapeutic targets for which inhibitors currently exist include the *WNT* signaling pathway, *MDM4*, *MET*, *VEGFA*, *MCL1*, *IDH1*, *TERT*, and various immune checkpoint proteins [46]. On the basis of these findings, the modification of genes related to tumor suppressors, oncogenes, those encoding the proteins expressed on the tumor cell surface, and the T-cell receptor to target the tumor, as well as genetic immunotherapy, have been tested in both basic and clinical research [22,23,24,46].

#### 2.2.1. Tumor Suppressor Genes

The abnormalities in tumor suppressor gene signaling pathways, including p53 and its negative regulator Mouse double minute 2 homolog (MDM2, are often found in HCCs [20,33,42]. Therefore, the restoration of tumor suppressor genes has been tested for its clinical applicability. Among them, p53 has been tested in several trials for HCC (NCT00003147, NCT02561546, NCT02509169, NCT02418988, and NCT02432963), as well as p53 gene vaccination for other liver tumors, including metastatic tumors from other organs (NCT02432963). The injection route includes the percutaneous, hepatic artery, and the combination with transarterial chemoembolization has also been tested and showed efficacy by improving the prognosis compared with Transarterial chemoembolization (TACE) monotherapy [47]. Administration of a dominant negative form of cyclin G1, also known as Rexin-G, using retroviral vector injection into the hepatic artery, has also been tested in the liver metastasis of colorectal tumors (NCT00035919) and has shown significant anti-tumor activity in pancreatic cancer [48]. At the basic level, other pro-apoptotic genes, such as Tumor necrosis factor (TNF)-related apoptosis-inducing ligand (TRAIL), have been tested to induce apoptosis in HCC cells. Adeno-associated virus (AAV)-human telomerase reverse transcriptase (hTERT)-TRAIL displayed cancer-specific cytotoxicity, and intratumoral administration of AAV-hTERT-TRAIL significantly suppressed tumor growth in a xenograft model [15,49,50].

#### 2.2.2. Oncogenes

A large number of the oncogenes related to HCC biology have been reported to date. The silencing of oncogenes has been tested in several studies using RNA interference (RNAi). Polo-like kinase 1 (PLK1) is a biomarker that can be used to evaluate the biological behavior and prognosis of the colorectal cancers [51] and is considered as an oncogene by cell cycle progression [52]. Indeed, siRNA against the PLK1 product using lipid nanoparticles (TKM-080301) has been tested (NCT01437007) for colorectal cancers with hepatic metastases by injecting into the hepatic artery [53]. Other oncogenes of *ZEH2, Smad4, osteopontin, reptin, Nob1, LDHA, SNAIL, GRK2, ATAD2,* and *STIM1* have also been tested for the interference effect in basic studies [23]. Other potential target oncogenes, including *Yes-associated protein* [54], which is a member of the Hippo signaling pathway contributing to organ size control and tumorigenesis, have emerged as attractive targets for cancer therapeutics for liver cancer [55,56]. *Survivin,* which is a member of the family of apoptosis inhibitory proteins, has increased expression in various cancers [57] and, as a result, has been considered as a potential biomarker and therapeutic target for HCC gene therapy [58].

Various oligonucleotide-based gene therapies are used to target tumor suppressor genes and oncogenes [59]. Oligonulceotide-based therapies include mRNA [60], siRNA [61,62,63], miRNA [64], and non-coding RNA [65,66,67,68,69]. A siRNA [61,62,63] is a 20–24 bp double-stranded RNA produced by Dicer enzyme from long dsRNA or small hairpin RNA that knocks down genes by cleaving target mRNA with a complementary sequence before translation. The combination of siRNA and N-acetylgalactosamine (GalNAc) has been used to increase the efficacy of siRNA to enter the cytoplasm through binding to the asialoglycoprotein receptor, which is highly expressed on hepatocytes [70]. miRNA [64] is a 22 bp non-coding RNA that functions in RNA silencing and post-transcriptional regulation of gene expression, and is derived from short stem-loop RNA [71]. It has been reported that expression of miR-122 in HCC with poorly differentiated, large-sized, and invasive characteristics is frequently decreased and, therefore, the increase of miR-122 levels in those HCCs, with or without anti-tumor agents, showed promising anti-tumor effects for HCC [64]. Long non-coding RNAs (lncRNA) [65,66,67,68,69] are a group of 200 nucleotides on protein coding RNA that play an important role in transcription, translation, and protein modification as oncogenes or tumor suppressor genes. They are also involved in different epigenetic cellular processes, such as proliferation, differentiation, migration, invasion, and anti-apoptosis. The lncRNAs have been used to predict prognosis, and zinc finger protein 385D antisense RNA 2 (ZNF385DAS2) is a lncRNA that has been used to predict the prognosis of patients with several types of cancer, including liver cancers [67], and can be a useful therapeutic target [69].

#### 2.2.3. Suicide Gene Therapy

Suicide gene therapy is based on the delivery of transgenes that convert prodrugs and are administered following gene delivery into cytotoxic metabolites and have shown anti-tumor effects [72]. The bystander effect of the cell–cell contact shows that cytotoxicity in the tumors cells neighboring the tumor cells is a characteristic of the therapy [72]. The most widely used combination of transgene and prodrug for HCC gene therapy is herpes simplex virus thymidine kinase (*HSV-tk*) and ganciclovir (GCV) [73,74,75]. Adenoviral vectors have been used to deliver *HSV-tk* injected either intravenously (NCT02202564, NCT00300521, and NCT03313596) or intratumorally (NCT00844623). Oncolytic virotherapy has also been reported for its anti-tumor effect [76] for various cancers including HCC and other liver tumors. Recently, oncolytic herpes simplex virus type-1 (HSV-1) has been tested for metastatic liver cancer from colorectal cancer (NV1020, NCT00012155) injected into the hepatic artery, and for HCC, other primary liver cancers, and metastatic liver tumors by administration via the hepatic artery (NCT01071941). The combinations of oncolytic virotherapy, other chemo-agents, and immune modifiers change the sensitivity of the tumor to the therapeutic options including immune checkpoint inhibition [77]. Thus, the suicide gene must be elicited in a tumor-specific manner using transcriptionally targeted retroviral replicating vectors [78], targeting genomic rearrangement in the tumor by genome-editing approach to insert the suicide gene [79]. One of the promising future targets includes diphtheria toxin A, an immunotoxin, which has been widely used in gene therapy for its roles in protein synthesis inhibition [80]. This gene has also been used in pancreatic cancer [81,82], ovarian cancer [83], glioblastoma, HCC [84], and bladder cancer [85] using various delivery methods including an integrase-deficient lentiviral vector [80] and plasmid DNA [82,84,85].

#### 2.2.4. Tumor Proteins

Glypican-3 (GPC-3) has also been tested to modify chimeric antigen receptor (CAR)-T-cells to treat HCC (NCT02715362, NCT03198546, and NCT02905188) administered through the hepatic artery, systemically, or by local injections. GPC-3 is a transmembrane heparan sulfate proteoglycan that regulates cell growth by tissue-dependent cellular signaling [86]; as its expression is increased in liver cancers, it has been used as a tumor maker and currently in ex vivo gene therapy to modify CAR-T to target HCC [87,88]. The alternative therapeutic option can be realizable in cases with p53-altered HCCs using aurora kinase A and the MYC complex based on results in xenograft models showing that p53-altered HCCs are hypersensitive with conformation-changed aurora kinase A [89]. The GPC-3-expressing T-cells have been tested for anti-tumor effects in pediatric liver cancers (NCT02932956) in combination with chemotherapy.

Alpha-fetoprotein (AFP) is one of the major tumor markers for HCC used in clinics [90] and are specific antigens presented on the cell surface [91]; thus, AFP is used for molecular targeting of CAR-T-cells for HCC (NCT03971747) in both ex vivo gene therapy and cancer vaccination (NCT00005629 and NCT03971747). With regard to cancer vaccination, intradermal injection of AFP peptide or adenoviral vector was used for HCC (NCT00093548). The safety and efficacy of AFP DNA prime and adenovirus boost immunization for HCC have also been tested [92].

Carcinoembryonic antigen (CEA) is useful for detecting recurrent metastatic colorectal cancers [93] as well as various CEA-related cell adhesion molecules [94]. As such, CEA has been used as a molecular target of CAR-T-cells in the treatment of liver metastases of colorectal cancers (NCT02416466, NCT02850536, and NCT00004178) and for developing T-cell therapy (NCT01373047) as an ex vivo gene therapy. These T-cells were administered via the hepatic artery, splenic vein, or veins. Further modification includes the administration of the vaccinia-CEA-mucin 1 triad of costimulatory molecules, and the TRICOM (PANVAC-V) vaccine has been tested for metastatic liver cancer from colorectal cancer (NCT00103142) [95].

Human epidermal growth factor receptor 2 (HER2) is a member of the epidermal growth factor family, and anti-HER2 treatment for HER2-positive breast cancer has shown promising efficacy [96]. Currently, a trial involving intravenous injection of an antibody drug conjugate targeting HER2-expressing cancers cells, including liver cancer and metastatic liver cancer, is ongoing (NCT03602079).

New York esophageal squamous cell carcinoma 1 (NY-ESO-1) is a cancer-testis antigen that is expressed in various cancer cells, and specific immune responses to it have been observed in various cancers [97]. NY-ESO-1 has been used in immune-based cancer therapy using genetically modified T-cells in clinics for NY-ESO-1 expressing solid tumors, including liver cancers (NCT02869217). Anti-NY-ESO-1 murine T cell receptor (TCR)-gene engineered lymphocytes have been administered intravenously for HCC and metastatic liver cancers from melanoma (NCT01967823).

#### 2.2.5. Genetic Immunotherapy

Local expression of cytokine expression genes has been tested for anti-tumor efficacy, as the systemic administration of cytokines may result in severe adverse events [98]. The cytokines include interferon-β expressed in an adenoviral vector injected intrapleurally for metastatic liver cancer (NCT00066404), expressed in a vesicular stomatitis viral vector injected intratumorally (NCT01628640) for advanced liver tumor, and interleukin-12 expressed in an adenoviral vector by intratumoral injection for liver metastasis from the breast cancer (NCT00301106) and for liver metastasis of colorectal cancer (NCT00072098). Autologous whole-cell tumor cell immunotherapy (FANG) has also been trialed (NCT01061840); this incorporates an intradermally injected plasmid encoding granulocyte macrophage colony-stimulating factor (GM-CSF) and a bifunctional short hairpin RNAi vector targeting furin convertase, thereby downregulating endogenous immunosuppressive transforming growth factors beta1 and beta2. Genetic immunotherapy also includes mRNA vaccination therapy; NCT03480152 is a clinical trial testing the efficacy of the anti-tumor effect of mRNA containing epitopes from immunogenic neoantigens, predicted neoantigens, and mutations in tumor suppressor or driver genes for HCC and metastatic liver tumors from colon cancer.

### 2.3. Gene Delivery Procedures

**Figure 1:**
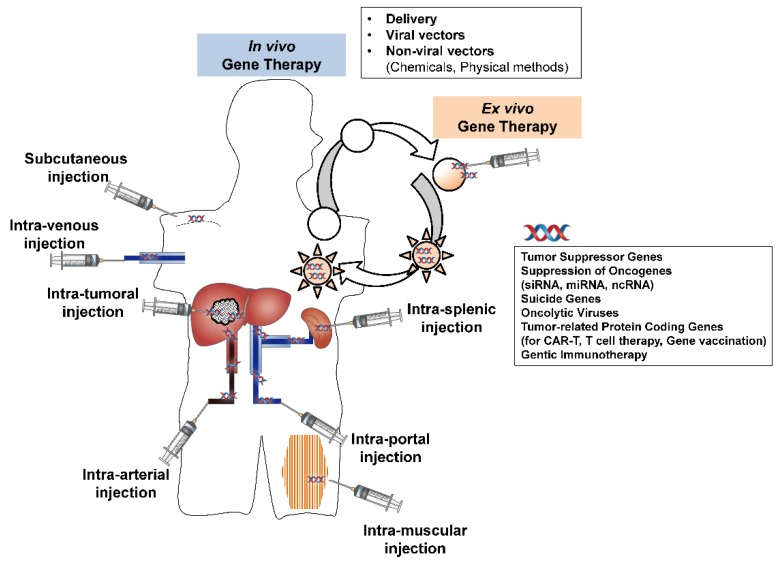
Schematic summary of gene therapy strategies for liver cancers. siRNA: small interfering RNA, miRNA: microRNA, ncRNA: non-coding RNA, CAR-T: chimeric antigen receptor-T-cell.

Studies focusing on gene delivery approaches for liver cancers have also been reported [23,99,100]. Gene delivery procedures can be classified into viral gene delivery, non-viral gene delivery using chemical compounds, and non-viral gene delivery using physical methods [101,102,103]. Various gene delivery procedures used in these studies are summarized in Table 1 and Figure 1 to understand the advantages and disadvantages of each procedure, which will help to develop the novel methods of delivery.

#### 2.3.1. Viral Gene Delivery

Clinically, more than 67% of gene therapy clinical trials have been conducted using viral vectors [28]. A virus-based gene delivery system represents a group of artificially made, replication-deficient viruses [104,105]; the most commonly used virus-based gene delivery systems are adeno-associated viral vectors [49,106], lentiviral vectors [105,107], adenoviral vectors [105,108], foamy viral vectors [109], herpes simplex viral vectors [110], oncoretroviral vectors [105], and cytomegaloviral vectors [111]. Among them, AAV vectors are often used; these are derived from the parvovirus and have a single-stranded DNA genome of approximately 4.7 kb. There are two genes in viral genome DNA, rep and cap, encoding seven major transcription units, Rep40, Rep52, Rep68, Rep78, VP1, VP2, and VP3 [112]. There are several serotypes of AAV, and it is worth noting that each serotype of AAV has a unique approach for infecting host cells. Single-stranded DNA is converted to the double-stranded vector genome from which the transgene is expressed. The target organ preference depends on the infectivity of wild serotype to those organs, and AAV-8 preferentially transduces into hepatocytes [113]; thus, AAV-8 is suitable for liver-targeted gene therapy intended for treatment of citrullinemia [114], hemophilia [115,116], alpha 1-antitrypsin deficiency [117], and viral hepatitis diseases [118].

The first gene therapy studies using AAV for HCC were reported by Su H, et al. using the HSV-tk gene driven by an AFP enhancer and the albumin promoter. They showed AFP-positive cell-specific tumor cell suppression that inspired a number of subsequent studies and trials [119]. In most of the studies, viral vector-mediated gene delivery to the liver was achieved via the hepatic artery [120,121], portal vein [122,123], bile duct [122], or by direct injection to the liver [122]. Interestingly, the clinical studies revealed the efficacy of positron emission tomography imaging the intratumoral injection of adenoviral vector for HCC [124]. Overall, however, viral vector-induced carcinogenesis and immunogenicity is currently a major hurdle for viral vector-mediated gene therapy.

#### 2.3.2. Non-Viral Gene Delivery Using Chemicals

Compared with viral vectors that employ their natural ability to transfer genes into cells, non-viral gene delivery systems use physical force or the cellular function of endocytosis to facilitate gene transfer to target cells [102,125]. Clinically, 21% of gene therapy clinical trials have been conducted using non-viral vectors [28]. The major challenge for non-viral vector-mediated gene delivery is its relatively low efficiency.

Non-viral vectors using chemicals include synthetic or natural compounds that are capable of forming complexes with plasmid DNA or gene coding fragments and facilitating intracellular gene transfer. Materials including lipids [126], polymers [127], proteins [128], and peptides [128] have been shown to be effective for gene delivery to tissues [129,130] and in HCC [131]. Non-viral vectors have been evaluated for gene therapy of a variety of liver diseases including hepatic fibrosis, viral hepatitis, and liver cancer [24]. Taking advantage of membrane receptors on hepatic stellate cells, liver-targeted gene delivery for hepatic fibrosis has been attempted using the mannose 6-phosphate/insulin-like growth factor-II receptor [132,133,134], integrins [135], high-affinity membrane receptor for retinol-binding protein, and the galactosyl receptor as targets [101]. Target-specific gene delivery is a desirable feature, and polymeric nanoparticles [131] targeting cancer-specific DNA have shown promising anti-tumor effects in vitro and in vivo [136]. A cationic solid lipid nanoparticle also showed effective inhibition of HCC growth by delivering shRNA for the *NURP* gene [137], and similarly, siRNA to PLK1 gene delivered by chitosan nanoparticles efficiently suppressed HCC cell growth both in vitro and in vivo [138]. Gold nanoparticles, which have been used as attractive chemical vehicles for the gene delivery [139], and are currently used for gene editing [140], have also been tested for their applicability in HCC gene therapy in vivo, owing to their ability to modify the surface to attach multiple ligands, their superior visibility, and their low cytotoxicity [141].

Recently, the integrins and heparan sulfate proteoglycans on hepatic stellate cells have been considered as receptors for exosomes [142]. Exosomes are small membranous vesicles released by most cells, including tumor cells that contain biological molecules, such as non-coding RNAs, and participate in regulating tumor development, metastasis, and drug resistance. Therefore, exosomal components have emerged as potential biomarkers, and exosomes can serve as natural vehicles to deliver non-coding RNA for treatment [69]. Exosomal components may be a next-generation non-viral delivery procedure, and further assessment is essential.

#### 2.3.3. Non-Viral Gene Delivery Using Physical Methods

Physical methods of gene delivery employ a physical force to overcome the membrane barrier of a cell. Compared with viral and chemical vector-mediated gene delivery, physical approaches do not involve any cytotoxic or immunogenic substances. Physical methods employed for gene delivery include needle injection, gene gun, electroporation, sonoporation, and hydrodynamic gene delivery [102]. Among these methods, sonoporation has shown potential to express pro-apoptotic genes in HCC cells in vitro [143] and deliver shRNA of frizzled-2 to suppress HCC in vitro [144]. Electroporation has been used for delivering TRAIL/Apo2L gene to induce apoptosis [145]; the IL-12 gene to induce immune responses to HCC [146,147]; more recently, to deliver mRNA into T-cells to develop specific T-cells for HCC immunotherapy [148]; and GPC-3 CAR-T-cells [149]. Magnetofection has also been used to deliver genes into HCC cell lines combined with ternary organic–inorganic hybrid nanocomposites containing deferoxamine-coated iron oxide nanoparticles, plasmid DNA, and branched polyethyleneimine [150]. Hydrodynamic gene delivery has been used for functional analysis of therapeutic genes and regulatory elements in rodents since its establishment in 1999 [151,152]. Efforts have been made in developing a clinically applicable procedure for hydrodynamic gene delivery to the liver. For instance, Kamimura et al. examined a catheter insertion technique to the hepatic lobular vein for site-specific, safe, and efficient gene delivery in pigs and dogs [153,154]. This procedure has shown therapeutic effect in liver fibrosis [155,156], and recently in HCC in mice driven by AKT over expression delivering a dominant negative form of heat shock transcription factor 1 [157]. The challenges for non-viral gene delivery are the lower efficiency of gene delivery compared with viral vectors, although target/tumor-specific gene delivery can be achieved better than viral vectors. The combination of these viral and non-viral gene delivery procedures might be effective, as evidenced in the recent reports showing that polyethyleneimine, ultrasound, and nanobubbles can effectively deliver shRNA in liver cancer [158].

To further extend the tumor-specific gene delivery, various gene delivery routes have been tested including intratumoral injection, intrasplenic injection, intra-arterial injection, intravenous injection, intraportal injection, intramuscular injection, subcutaneous injection, oral injection, and liver incisal margin injection [23]. In addition, promoter selective gene expression is another method of HCC-specific gene delivery, and the AFP promoter has been used to induce HSV-tk gene using the AAV vector [119].

### 2.4. Clinical Trials

Conventional diagnostic strategies, such as computed tomography and biopsies, as well as the development of molecular biology and techniques such as new generation sequencing and single cell analyses, have significantly contributed to the understanding of the pathogenesis of liver cancer and provided novel therapeutic options. On the basis of the progress of gene therapy described above, various clinical trials are ongoing [28] and gene therapy has become a realistic treatment option for a wide variety of cancers [25,26]. 

#### 2.4.1. Ongoing Clinical Trials for Gene Therapy of Liver Cancers

The summary of the results of several completed and ongoing clinical trials for HCC is shown in Table 2 and Supplementary Table S1. Building on preclinical studies, several clinical trials have been conducted to evaluate gene therapy for liver cancers [53,73,74,87,92,95,124,159,160,161,162,163,164,165,166,167]. A summary of these studies as of October 2019 can be found in Table 2 and Supplementary Table S1. The description of genes used has been outlined in the previous sections. Adenoviruses [73,124,165], oncolytic herpes simplex viruses (NCT00012155) [74], retroviruses, plasmids, and synthetic vectors were used to deliver tumor suppressor genes, suicide genes (NCT00844623) [73,124], cytokine genes, or antigens (peptides) for the anti-tumor effect and for cancer vaccination (NCT03480152) [87,92,159,160,161]. Moreover, T-cells (NCT01967823) [162,163,164] and CAR-T-cells (NCT03198546) [87] have been used in ex vivo gene therapy to induce tumor cell-specific immune responses. These vectors and cells have been delivered by percutaneous, intrahepatic arterial injection, intravenous injection, intrasplenic venous injection, intratumoral injection, intramuscular injection, intrapleural injection, and intradermal injection. In addition, some studies have combined gene delivery with the traditional therapeutic option of transarterial embolization (TAE) for HCC injecting genes into the embolized artery following TAE. Among the 34 clinical trials in which information is registered, only one was a phase III study, and the remaining studies were phase I or II. The phase III trial was a multicenter randomized controlled trial of adenovirus-mediated adjuvant gene therapy for patients with HCC who received liver transplantation. The study compared the effect of liver transplantation plus adenovirus-mediated suicide gene therapy versus transplantation in advanced primary HCC (NCT03313596). To date, 180 cases were enrolled, and the trial will be completed in December 2019. The genes transferred include genes encoding p53, TK of herpes simplex virus (HSV-tk), AFP (NCT00093548) [92], interferon-beta (NCT00066404) [165], interleukin-12, dominant negative form of cyclin G1, HER-2, GM-CSF (NCT01061840) [166,167], CEA, glypican 3-specific chimeric antigen (NCT03198546) [87], mRNA containing epitopes from immunogenic neoantigens, predicted neoantigens and mutations in tumor suppressor or driver genes [159,160,161], and autoimmunogenic cancer/testis antigen New York esophageal squamous cell carcinoma 1 (NY-ESO-1) [162,163,164]. The phase II gene vaccination study has been tested for its anti-tumor effect in metastatic liver cancer genes (NCT00103142). In addition, other oligonucleotides, including short hairpin RNA combined with a GM-CSF expressing plasmid [166,167], as well as short interfering RNA against polo-like kinase 1 (PLK-1) gene products carried by lipid nanoparticles (NCT01437007) [53], have also been tested. While detailed information is available for 34 studies, only a few results have been reported to date and the remainder of the study is mostly under assessment (Table 2 and Supplementary Table S1). NCT00844623 showed the safety and the potential anti-tumor efficacy of HSVtk gene using adenoviral vector in phase 1 study [73]. Further, related to NCT01967823, TCR-based gene therapies against NY-ESO-1 showed anti-tumor effect in the metastatic melanoma tumors [164]. NCT00012155 reported its results, showing that the NV1020, oncolytic HSV-1, can be safely administered into the hepatic artery of the cases with metastatic colorectal carcinoma to the liver [74]. A phase I trial of hIFN-beta gene using the adenoviral vector for the metastatic liver tumors (NCT0006640) showed safety and disease stability of the approach, although the rapid development of antibody to the vector was revealed to be the issue [165]. Furthermore, although the clinical trial (NCT00093548) was withdrawn, gene vaccination using AFP gene showed better progression-free survival [92]. The phase 1 trial of bi-shRNAi(furin)/GMCSF DNA/autologous tumor cell vaccine (NCT01061840) showed its safety [167]. A hepatic arterial infusion of lipid nanoparticles containing siRNA against the PLK1 gene product showed potential usefulness of the product for the metastatic liver tumors [53]. NCT00103142 showed the anti-tumor effect for the metastatic liver tumors of gene vaccination using autologous dendritic cells in the phase 2 study. Although NCT3480152 has a background of basic studies showing the importance of TCR affinity and tumor specific CD4^+^ T cells in tumor immunotherapy for HCC and metastatic liver tumors [159], and NCT03198546 has a background of successful suppression of HCC growth in the basic research [87], no clinical results have been reported to date for these trials.

#### 2.4.2. Ongoing Clinical Trials for Gene-Based Diagnosis

The summary of clinical trials for the molecular-based diagnosis and to determine the genetic background relating to chemosensitivity are shown in Table 3. The investigation of molecular tumor features is essential in order to efficiently translate the results of basic research. For this purpose, genetic analyses of liver tissues from patients with HCC for genomic medicine have provided important information about tumor initiation, progression, and chemosensitivity [168]. The findings from these studies can be used to develop personalized gene-based therapy and genome-based diagnosis in the tumor; therefore, various clinical trials to determine the disease activity and sensitivity to the specific therapy are ongoing [169,170,171,172,173]. One of the trials bridged into the phase 1/2 trial is NCT03480152, examining the effect of mRNA cancer vaccine, delivering mRNA containing epitopes from immunogenic neoantigens, predicted neoantigens, and mutations in tumor suppressor or driver genes, by intramuscular injection [159,160,161]. A summary of these studies as of October 2019 can be found in Table 3. Among the 14 clinical trials in which information is registered, only one was a phase IV study, and the remaining studies were phase I or II. The phase IV study assessed the mechanism of sorafenib resistance in patients with HCC [169].

These studies include a microarray analysis of gene expression patterns in liver tumors to determine new tumor and treatment markers (NCT00373737); screening of the methylation phenotype of liver cancer to predict the prognosis (NCT01786980); analysis of different gene expression patterns in liver cancer and the blood to determine genes that are expressed in both circulating white blood cells and the liver of patients with varying degrees of liver damage of different causes (NCT00160940); a genotype-guided dosing analysis of mFOLFIRINOX for primary and metastatic liver cancers (NCT01643499); phase II molecular analysis to assess how well the treatment, directed by genetic testing, works in patients with solid tumors or lymphomas that have progressed following at least one line of standard treatment, or for which no agreed treatment approach exists (NCT02465060); an analysis of the molecular mechanism of sorafenib resistance in HCC patients assessed by gene expression profiles (NCT02733809) [169]; a phase I/II study to determine fibroblast growth factor receptor (FGFR) genetic alterations treated with novel FGFR inhibitor (ARQ-087) (NCT01752920) [170]; a phase I study to determine genetic alteration of the proto-oncogene MET in patients with solid tumors, including liver cancer treated with a novel MET/CSF1R/SRC inhibitor, TPX-0022 (NCT03993873); an investigation of vascular endothelial growth factor receptor (VEGFR), promoting cell growth and metastasis in HCC (NCT01892072); an assessment the impact of IL-28B rs12979860 and rs4803217 gene polymorphisms on hepatitis C virus (HCV)-related HCC (NCT02507882) [171]; analyses of the expression of a specific set of genes and of tumor antigens in cancer tissue from patients with HCC (NCT00858000); an assessment of matrix metalloproteinase-1 genotype polymorphism as a risk factor for HCV-related HCC (NCT03722628) [172]; determination of the role of circulating tumor cells as biomarkers of prognosis and predictors of efficacy of drug therapy for patients with HCC (NCT01930383); and a phase II study comparing the efficacy and safety of SOR versus infusional 5-fluorouracil (5-FU) in HCC based on the information of pERK concentration, phospho VEGFR concentration, plasma proteomics, and gene expression (NCT00619541) [173] (Table 3). While the detailed information is available for 14 studies, only a few results have been reported to date and the remainder of the study is mostly under assessment (Table 3). NCT01752920 showed the results of anti-tumor effect and safety of Derazantinib (ARQ 087) for unresectable intrahepatic cholangiocarcinomas with FGFR genetic alterations [170]. In addition, the phase 2 trial of the combination of SOR with 5-FU showed an encouraging disease control rate and overall survival [173]. Although NCT02733809 has a background of basic studies suggesting the molecular pathways blocked by the sorafenib [169], and NCT03722628 has evidence that the genetic variations of *MMP-11* gene is related to the progression of HCC and can be a biomarker [172], no clinical results have been reported to date for these trials. Further studies are necessary to analyze gene expression related to chemosensitivity and toxicity, and to develop a standard and safe chemotherapy for HCC.

## 3. Recent Progress

CAR-T cells have been developed, and based on the success of treating hematological malignancies, they have become one of the most promising therapeutic options, even in solid tumors [174,175,176]. However, the lack of specific antigens in the solid tumors, especially liver cancer with heterogeneous tumor cells, limited penetration of the CAR-T-cells into tumor sites, and immunosuppressive tumor microenvironment are major obstacles to apply this method for HCC treatment. Most CARs use a single-chain variable fragment constructed from the variable heavy and light chains of a tumor-associated antigen-specific monoclonal antibody as the extracellular antigen recognition domain; a ligand or receptor can also be used. T-cells are collected from the patient and activated using anti-CD3 and IL-2, genetically modified, and expanded in vitro. The developed cells are then evaluated to ensure CAR expression, and infused to the patients. Currently, glypican-3 (GPC-3) has been tested to modify CAR-T-cells to treat HCC (NCT02715362, NCT03198546, and NCT02905188), as described [87], as well as for colorectal cancers (NCT02416466, NCT02850536, and NCT00004178). Similarly, T-cell-based ex vivo gene therapy has been tested (NCT01373047, NCT02932956, and NCT02869217).

Genome-editing technologies, including ZFN, TALEN, and Cas9 systems, have significantly broadened the ability to edit the genomic DNA in vitro, and even in vivo [29,32,177,178]. Delivery of in vitro-transcribed mRNA-mediated delivery of nucleases has various applications and future prospects of genome editing in research and clinical trials [179]. Recent progress showed the significance of combining these viral and non-viral gene delivery approaches for therapeutic genome editing. For example, it has been reported that the lipid nanoparticle-mediated delivery of Cas9 mRNA with AAVs encoding a sgRNA and a repair template to induce repair of a disease gene in adult animals showed successful genome editing and therapeutic effect [32]. Further efforts are necessary to develop the safe and effective delivery of the CRISPR/Cas9 system [180].

## 4. Conclusions

Among the various diseases affecting liver function, liver cancer is one of the leading causes of cancer-related deaths worldwide. Although conventional therapeutic options of surgery, ablation, chemoembolization, systemic chemotherapy, and molecularly targeted agents are partly effective for HCC, they are not sufficient for advanced-stage liver cancer in terms of efficacy. Therefore, the novel therapeutic option is an unmet need because of the heterogeneity of the tumors. On the basis of the development of genetic information, molecular biology, and analysis methodologies, gene therapy has shown promising anti-tumor effects in basic research and recent clinical trials. To further extend the applicability of gene therapy and the basic research in the field, we have carefully reviewed the genes and delivery methods, and summarized the currently ongoing clinical trials as of October 2019. Although further studies are essential to improve the efficacy and safety, with recent advances in promising technologies, such as gene editing by CRISPR/Cas9, CAR-T therapy, and the development of delivery systems armed with personal genomic information, gene therapy for liver cancer could improve the prognosis of patients with liver cancer.

## Figures and Tables

**Table 1:** Gene delivery methods used in clinical and preclinical stages.

Gene Transfer Methods	Genetic Materials (Cloning Capacity)/ Functional Component	Advantages		Disadvantages
Viral Vectors				
**Retroviral Vectors**				
Oncoretrovirus	Single stranded RNA (8 kb)	High transduction efficiency	Infect to dividing cells	Random integration Low efficiency of purification
Lentivirus	Single stranded RNA (8 kb)	High transduction efficiency Sustained gene expression Low immune response	Infect to dividing and non-dividing cells	Random integration Low efficiency of purification
Foamy virus	Single stranded RNA (9.2 kb)	High transduction efficiency Sustained gene expression No expression of viral proteins Low immune response	Infect to dividing cells Form a stable transduction intermediate in non-dividing cells	Random integration Low efficiency of purification
**Adenoviral Vectors**				
First generation adenovirus (FGAd)	Double stranded DNA (8–10 kb)	High transduction efficiency	Infect to dividing and non-dividing cells	Transient expression Host innate immune response Complicated vector production
Helper-dependent adenovirus (HDAd)	Double stranded DNA (~37 kb)	Large insert size Essentially no integration	Infect to dividing and non-dividing cells	Transient expression Host innate immune response Complicated vector production
**Adeno-associated virus**	Single stranded DNA (4–5 kb)	Non pathogenic Sustained gene expression Mainly no integration Low immune response	Infect to dividing and non-dividing cells	Integration may occur Small capacity of transgene Transient expression Complicated vector production
**Herpes simplex virus**	Double stranded DNA (~30 kb)	Large insert size No integration Sustained gene expression	Infectivity to nervous system	Transient expression Low transduction efficiency
** Non-viral Vectors (Chemicals)**				
**Cationic lipids**	Cationic charge, hydrophobic domain	High efficiency in vitro, ease to prepare		Low efficiency in vivo, acute immune response
**Cationic polymers**	Cationic charge, polymer	Highly effective in vitro, ease to prepare		Toxic to cells, acute immune response
**Proteins**	Natural or chemically modified proteins in cationic nature	Highly effective in vitro, less toxic, can be target specific		Low activity in vivo
**Peptides**	Lysine or arginine residues in peptides	Highly effective in vitro, less toxic, can be target specific		Low activity in vivo
**Non-viral Vectors (Physical Methods)**				
**Needle injection**	Mechanic force	Simple		Low efficiency, expression limited to needle track
**Gene gun**	Pressure	Ease, Good efficiency		Limited to target area, need surgical procedure for internal organ
**Electroporation**	Electric pulse	High efficiency		Tissue damage, limited target area, need surgical procedure for internal organ
**Sonoporation**	Ultrasound	Simple, can be site-specific		Low efficiency, tissue damage
**Magnetofection**	Magnetic field	Site specific		Low efficiency, limited target area, need surgical procedure for internal organ
**Hydrodynamic delivery**	Hydrodynamic pressure	Simple, high efficiency in vivo, site specific		Need catheter insertion technique in large animals
**Immunotherapy**	CAR-T, T cells	Antigen-specific		Require ex vivo cell culture Poorly effective for solid tumors
**Gene Vaccination**	Antigen-pulsed dendritic cells	Ease to prepare less toxic, ease to administer		Low efficacy

**Table 2:** Summary of ongoing clinical trials for gene therapy for liver cancers.

No	NCT Number	Types of Liver Tumors	Gene/Antigen	Types of Gene	Vectors or Cells	Intervention	Route of Administration	Phase	Current Status and Results	Ref.
1	NCT00003147	HCC	p53		Adenoviral Vector	Ad5CMV-p53 gene	Percutaneous injection	1	Terminated No results available	
2	NCT01071941	Primary Liver Cancers	Oncolytic Virus	Oncolytic Virus	Herpes simplex virus 1	rRp450	Hepatic arterial injection	1	Recruiting No results available Estimated Completion Date: July, 2020	
		Metastatic Liver Tumors								
3	NCT00844623	HCC	HSVtk	Suicide	Adenoviral vector	TK99UN (adenoviral vector containing TK)	Intratumoral injection	1	Completed Results partly reported.	[73,124]
4	NCT02202564	HCC	HSVtk	Suicide	Adenoviral vector	LT ADV-TK ganciclovir	Intravenous infusion	2	Completed No results reported to date	
5	NCT02561546	HCC	p53	Tumor suppressor	Recombinant adenoviral vector	p53 gene therapy TAE	Hepatic arterial injection following TAE	2	Not yet recruiting	
6	NCT00300521	HCC	HSVtk	Suicide	Adenoviral vector	ADV-TK	Intravenous infusion	2	Completed No results reported to date	
7	NCT00004178	Primary Liver Cancers Metastatic Liver Tumors	CEA	Tumor-related Protein Coding	T Cells Modified with Chimeric Anti-CEA Immunoglobulin-T Cell Receptors (IgTCR) in Adenocarcinoma	Therapeutic autologous lymphocytes	Intravenous infusion	1	Completed No results reported to date	
8	NCT03313596	HCC	HSVtk	Suicide	Adenoviral vector	ADV-TK LT	Intravenous infusion	3	Recruiting No results available Estimated Completion Date: Dec, 2019	
9	NCT03480152	Primary Liver Cancers	mRNA containing epitopes from immunogenic neoantigens	Tumor-related Protein Coding	mRNA vaccine	NCI-4650, a mRNA-based, Personalized Cancer Vaccine	Intramuscular injection	1/2	Terminated Related results partly reported	[159,160,161]
		Metastatic Liver Tumors	mRNA containing epitopes from immunogenic predicted neoantigens							
			mRNA containing epitopes from immunogenic mutations in tumor suppressor or driver genes							
10	NCT01967823	HCC Metastatic Liver Tumors	NY-ESO-1	Tumor-related Protein Coding	Anti-NY ESO-1 Murine TCR-Gene Engineered Lymphocytes	Anti-NY ESO-1 mTCR PBL Cyclophosphamide Fludarabine Aldesleukin	Intravenous infusion	2	Recruiting No results available Estimated Completion Date: July, 2028	[162,163,164]
11	NCT02509169	HCC	p53	Tumor suppressor	Recombinant adenoviral vector	TAE plus P53 gene TAE	Hepatic arterial injection following TAE	2	Recruiting No results available	
12	NCT02932956	Pediatric Primary Liver Cancers	GPC-3	Tumor-related Protein Coding	CAR T cells	GAP T cells Cytoxan Fludara		1	Recruiting No results available Estimated Completion Date: Feb, 2037	
13	NCT00012155	Metastatic Liver Tumors	Oncoytic Virus	Oncoytic Virus	oncolytic herpes simplex virus type-1(HSV-1)	NV1020, oncolytic herpes simplex virus type-1 (HSV-1)	Hepatic arterial injection	1	Completed Results partly reported	[74]
14	NCT00066404	Metastatic Liver Tumors	Interferon-beta	Genetic Immunotherapy	Adenoviral vector	recombinant adenovirus-hIFN-beta	Intrapleural injection	1	Active, not recruiting Results partly reported	[165]
15	NCT00035919	Metastatic Liver Tumors	Dominant Negative Cyclin G1	Tumor suppressor	Retroviral Vector	Mx-dnG1 Retroviral Vector	Hepatic arterial infusion	1/2	Withdrawn	
16	NCT00005629	Primary Liver Cancers Metastatic Liver Tumors	AFP	Tumor-related Protein Coding	AFP peptide	AFP gene hepatocellular carcinoma vaccine	Intradermal injection	1/2	Completed No results reported to date	
17	NCT02905188	HCC	GPC-3	Tumor-related Protein Coding	CAR T cells	GLYCAR T cells	Intravenous infusion	1	Recruiting No results available Estimated Completion Date: Oct, 2036	
18	NCT00093548	HCC	AFP, GM-CSF	Tumor-related Protein Coding	Adenoviral vector	Vaccination AFP plasmid DNA vaccine GM-CSF plasmid DNA hepatocellular carcinoma vaccine adjuvant	Intramuscular injection/Intradermal injection	1/2	Withdrawn	[92]
19	NCT01628640	Primary Liver Cancers Metastatic Liver Tumors	Interferon-beta	Genetic Immunotherapy	Vesicular Stomatitis Virus	Recombinant Vesicular Stomatitis Virus-expressing Interferon-beta	Intratumoral Injection	1	Active, not recruiting Estimated Completion Date: June, 2025	
20	NCT03602079	HCC CCC Metastatic Liver Tumors	HER-2	Tumor-related Protein Coding	Antibody Drug Conjugate (ADC)	A166, an Antibody Drug Conjugate (ADC) targeting HER2 expressing cancer cells.	Intravenous infusion	1/2	Recruiting No results available Estimated Completion Date: May, 2021	
21	NCT02416466	Metastatic Liver Tumors	CEA	Tumor-related Protein Coding	CAR-T cells	anti-CEA CAR-T cells	Hepatic arterial infusion	1	Completed No results reported to date	
22	NCT02869217	NY-ESO-1 Expressing Liver Cancers in HLA-A2 Positive Patients Metastatic Liver Tumors	NY-ESO-1	Tumor-related Protein Coding	NY-ESO-1 Specific TCR Gene Transduced Autologous T Lymphocytes	TBI-1301 (NY-ESO-1 Specific TCR Gene Transduced Autologous T Lymphocytes) Cyclophosphamide	Infusion	1	Recruiting No results available Estimated Completion Date: June, 2020	
23	NCT01061840	Primary Liver Cancers Metastatic Liver Tumors	rhGMCSF and bi-shRNAfurin from the Vigil™ plasmid	Tumor-related Protein Coding	plasmid	Vaccination	Intradermal injection	1	Completed Results partly reported	[166,167]
24	NCT01437007	Metastatic Liver Tumors from Colorectal, Pancreas, Gastric, Breast, and Ovarian Cancers	siRNA Against the PLK1	Oncogene suppression	Lipid Nanoparticles	TKM-080301	Hepatic arterial infusion	1	Completed Results partly reported	[53]
25	NCT03971747	HCC	AFP	Tumor-related Protein Coding	T Cell	AFP Specific T Cell Receptor T Cells	Intravenous infusion	1	Not yet recruiting	
26	NCT02418988	HCC	p53	Tumor suppressor	Recombinant adenoviral vector	TACE plus rAd-p53 artery injection TACE	Injected into the embolization artery.	2	Recruiting No results available	
27	NCT02850536	Metastatic Liver Tumors from Colorectal, Pancreas, Gastric, Breast, and Ovarian Cancers	CEA	Tumor-related Protein Coding	CAR-T cells	anti-CEA CAR-T cells	Hepatic arterial infusion or splenic vein	1	Active, not recruiting Estimated Completion Date: Dec, 2019	
28	NCT01373047	Metastatic Liver Tumors from Colorectal, Pancreas, Gastric, Breast, and Ovarian Cancers	CEA	Tumor-related Protein Coding	T cell	anti-CEA 2nd generation designer T cells	Hepatic arterial infusion or splenic vein	1	Completed No results reported to date	
29	NCT02432963	Adult Solid Neoplasm	p53	Tumor suppressor	Modified vaccinia virus	Vaccination		1	Active, not recruiting Estimated Completion Date: Feb, 2020	
30	NCT02715362	HCC	GPC3	Tumor-related Protein Coding	CAR-T cells	TAI-GPC3-CART cells	Hepatic arterial infusion	1/2	Recruiting No results available	
31	NCT00301106	Metastatic Liver Tumors from Colorectal, Pancreas, Gastric, Breast, and Ovarian Cancers	Interleukin-12	Genetic Immunotherapy	Adenoviral Vector	adenovirus-mediated human interleukin-12	Intratumoral Injection	1	Terminated No results available	
32	NCT00072098	Metastatic Liver Tumors from Colorectal, Pancreas, Gastric, Breast, and Ovarian Cancers	Interleukin-12	Genetic Immunotherapy	Adenoviral Vector	adenoviral vector-delivered interleukin-12	Intratumoral Injection	1	Terminated No results available	
33	NCT03198546	HCC	GPC3	Tumor-related Protein Coding	CAR-T cells	GPC3 targeting CAR-T cells	Systemic or local injections	1	Recruiting No results available Estimated Completion Date: Aug, 2022	[87]
34	NCT00103142	Metastatic Liver Tumors from Colorectal, Pancreas, Gastric, Breast, and Ovarian Cancers	Vaccinia-Carcinoembryonic antigen (CEA)-mucin 1 (MUC-1)- Triad of costimulatory molecules TRICOM vaccine (PANVAC-V)	Tumor-related Protein Coding	Autologous dendritic cells	Vaccination		2	Completed Results available	[95]

HCC, hepatocellular carcinoma; CCC, cholangiocellular carcinoma; TACE, transarterial chemoembolization; TAE, transarterial embolization; HSVtk, thymidine kinase of herpes simplex virus; NY-ESO-1, New York esophageal squamous cell carcinoma 1; GPC-3, Glypican-3; LT, liver transplantation.

**Table 3:** Summary of ongoing clinical trials for gene-based diagnosis.

No	NCT Number	Official Title	Brief Summary	Types of Liver Tumors	Phase	Enrollment	Current Status and Results	Ref.
1	NCT00373737	Microarray Analysis of Gene Expression in Liver Tumors	This study aims to study the gene expression profiles of liver tumors to help us understand their biology, and to find new tumor and treatment markers for liver cancer.	Liver Cancer	Not Applicable	300	Completed No results reported to date	
2	NCT01786980	The Methylation Phenotype Screening and Determination Mode Study of Liver Cancer Prognosis Related Gene	This study aimed to obtain the important factors affecting liver cancer prognosis, survival, recurrence and metastasis in order to be able to find and establish the effective prognostic evaluation method by analyzing clinical information combining the information of gene chip, methylation chip and flow cytometry to carry out comprehensive researches on liver cancer cell genetics, epigenetics, stem cells and tumor microenvironment changes.	HCC	Not Applicable	300	Completed No results reported to date	
3	NCT00160940	Differential Gene Expression in Liver Tissue and Blood From Individuals With Chronic Viral Hepatitis With or Without a Complicating Hepatoma or Autoimmune Liver Disease	This study aimed to find the genes that are expressed in both the circulating white blood cells and the liver of patients, using differential gene expression analysis, with varying degrees of liver damage of different causes with or without liver cancers.	Primary Liver Cancers Metastatic Liver Tumors	Not Applicable	200	Recruiting No results available	
4	NCT01643499	A Genotype-guided Dosing Study of mFOLFIRINOX in Previously Untreated Patients With Advanced Gastrointestinal Malignancies	This study aimed to determine the dose of a chemotherapy drug (irinotecan) in 1st cycle in each of two UGT1A1 genotype groups (*1*1, *1*28) using genotype-guided dosing, that can be tolerated as part of a combination of drugs.	HCC CCC Metastatic Liver Tumors	1	79	Active, not recruiting Estimated Completion Date: Apr, 2019	
5	NCT02465060	Molecular Analysis for Therapy Choice (MATCH)	This phase II MATCH trial aimed to study how well treatment that is directed by genetic testing works in patients with solid tumors or lymphomas that have progressed following at least one line of standard treatment or for which no agreed upon treatment approach exists.	Primary Liver Cancers Metastatic Liver Tumors	2	6452	Recruiting No results available Estimated Primary Completion Date: June, 2022	
6	NCT02733809	Mechanism of Sorafenib Resistance in Patients With Advanced Hepatocellular Carcinoma	This study aimed to clarify the hypothesis that resistant tumor may be due to genetic mutations and/or other alternative pathways that could be the reason to overcome the SOR and still proliferate by analyzing the gene expression profiling signature (a set of dysregulated genes) for molecular classification, diagnosis, and prognosis of several types of cancers.	HCC	4	40	Recruiting No results available Estimated Completion Date: Dec, 2024	[169]
7	NCT01752920	A Phase 1/2 Study of ARQ 087 in Adult Subjects With Advanced Solid Tumors With FGFR Genetic Alterations, Including Intrahepatic Cholangiocarcinoma With FGFR2 Gene Fusion	This open-label, Phase 1/2, dose escalation and signal finding study aimed to clarify the effect of Derazantinib (ARQ 087), multi-kinase inhibitor designed to preferentially inhibit the FGFR family of kinases, in the cases with cholangiocarcinoma with FGFR2 gene alterations.	CCC	1/2	119	Completed Results partly reported	[170]
8	NCT03993873	A Phase 1, Open-Label, Multi-Center, First-in-Human Study of the Safety, Tolerability, Pharmacokinetics, and Anti-Tumor Activity of TPX-0022, a Novel MET/CSF1R/SRC Inhibitor, in Patients With Advanced Solid Tumors Harboring Genetic Alterations in MET	A phase 1, first-in-human, open-label study to determine the safety, tolerability, PK, and preliminary efficacy of the novel MET/CSF1R/SRC inhibitor TPX-0022 in adult subjects with advanced solid tumors harboring genetic alterations in MET. The study will proceed in three parts: a dose-escalation, a food effect, and dose-expansion.	Advanced Solid Tumor Metastatic Solid Tumors	1	120	Recruiting No results available Estimated Completion Date: Nov, 2023	
9	NCT01892072	VEGF Signaling Promotes Cell Growth and Metastasis in Hepatocellular Carcinoma in a VEGF Receptor Mediated Pathway	This study aimed to examine the VEGF signaling in HCC cell lines and its mechanism in HCC growth, proliferation and apoptosis.	HCC	Not Applicable	50	Active, not recruiting	
10	NCT02507882	Impact of IL-28B rs12979860 and rs4803217 Gene Polymorphisms Associated With miRNAs Deregulation on HCV-related Hepatocellular Carcinoma	This study aimed to determine through investigating a cohort of 405 patients, whether IL28B rs12979860 and rs4803217 polymorphisms are associated to the risk of HCC in chronic hepatitis C patients.	HCC	Not Applicable	405	Not yet recruiting	
11	NCT00858000	Analysis of the Incidence of Expression of a Specific Set of Genes and of Tumor Antigens in Cancer Tissue From Patients With Hepatocellular Carcinoma	This study aimed to analyze the expression of specific markers in HCC and tumor-related antigens to develop new approaches to treat this type of cancer with genetic immunotherapy.	HCC	Not Applicable	30	Completed No results reported to date	
12	NCT03722628	The Assessment of Matrix Metalloproteinase-1 Genotypes Polymorphism as a Risk Factor for Hepatocellular Carcinoma in Chronic Hepatitis C Patients With Liver Cirrhosis	This study aimed to assess whether the Matrix Metalloproteinase-1 genotypes polymorphism can be a risk factor for HCC in chronic hepatitis C patients with liver cirrhosis.	HCC	Not Applicable	200	Not yet recruiting	[172]
13	NCT01930383	Circulating Tumor Cells as Biomarkers of Prognosis and Predictors of Efficacy of Drug Therapy for Patients With Hepatocellular Carcinoma	This study aimed to explore the clinical value of correlation between circulating tumor cells numbers and other clinical characteristics in HCC patients with different stages.	HCC	Not Applicable	150	Recruiting No results available	
14	NCT00619541	Phase II Study of Sorafenib (Bay 43-9006) and Infusional 5-Fluorouracil in Advanced Hepatocellular Carcinoma.	The purpose of this study is to use SOR + 5-FU to evaluate activity, efficacy, safety, pharmacodynamics and pharmacokinetics in patients with advanced HCC.	HCC	2	46	Completed Results partly reported	[173]

HCC, hepatocellular carcinoma; FOLFIRINOX regimen A regimen consisting of leucovorin calcium, fluorouracil, irinotecan hydrochloride, and oxaliplatin; SOR, sorafenib; FGFR, fibroblast growth factor receptor; CCC, cholangiocellular carcinoma.

## Supplementary Materials

The following are available online at https://www.mdpi.com/2072-6694/11/12/1865/s1, Table S1 Full summary of ongoing clinical trials for gene therapy for liver cancers.
