# Supplementary material for: Gene Therapy for Liver Cancers: Current Status from Basic to Clinics
Source: Cancers (Basel). 2019 Nov 25;11(12):1865. doi: 10.3390/cancers11121865 (PMC6966544; doi:10.3390/cancers11121865)
Supplement: Supplementary file 1 [file cancers-11-01865-s001.pdf]

cancers

MDPI

Gene Therapy for Liver Cancers: Current Status from Basic to Clinics

Kenya Kamimura *, Takeshi Yokoo, Hiroyuki Abe, and Shuji Terai

Supplementary Table S1. Full summary of ongoing clinical trials for gene therapy for liver cancers.

No	NCT Number	Official Title	Brief Summary	Types of Liver Tumors	Gene/ Antigen	Types of Gene	Vectors or Cells	Intervention	Route of Administration	Phase	Enrollment	Current Status and Results	Ref.
1	NCT00003147	Phase I Study of Percutaneous Injections of Adeno-Virus p53 Construct (ADENO-p53) for Hepatocellular Carcinoma	This study aimed to study the effectiveness of gene therapy with the p53 gene in treating patients who have cancer of the liver that cannot be surgically removed. Inserting the p53 gene into a person's tumor may improve the body's ability to fight liver cancer.	HCC	p53		Adenoviral Vector	Ad5CMV-p53 gene	Percutaneous injection	1	30	Terminated No results available	
2	NCT01071941	rRp450-Phase I Trial in Liver Metastases and Primary Liver Tumors	This study aimed to determine the safety of rRp450 and the highest dose of this agent that can be given to people safely.	Primary Liver Cancers	Oncolytic Virus	Oncolytic Virus	Herpes simplex virus 1	rRp450	Hepatic arterial injection	1	40	Recruiting No results available Estimated Completion Date: July, 2020	
				Metastatic Liver Tumors									
3	NCT00844623	Phase I Clinical Trial of Gene Therapy for Hepatocellular Carcinoma by Intratumoral Injection of TK99UN (An Adenoviral Vector Containing the Thymidine Kinase of Herpes Simplex Virus)	The study aimed to evaluate the intratumoral injection of defective adenovirus containing HSVtk (the thymidine kinase of herpes simplex virus), in patients with advanced HCC that were not amenable to curative therapy.	HCC	HSVtk	Suicide	Adenoviral vector	TK99UN (adenoviral vector containing TK)	Intratumoral injection	1	10	Completed Results partly reported.	[73,124]
4	NCT02202564	Preliminary Results for the Double-dose Adenovirus-mediated Adjuvant Therapy Improving Outcome of Liver Transplantation in Patients with Advanced Hepatocellular Carcinoma	This study aimed to clarify the anti-tumor effect of adjuvant therapy of ADV-TK following liver transplantation for the patients with tumors >5 cm in diameter, regardless of involvement in the intrahepatic and extrahepatic portal branches.	HCC	HSVtk	Suicide	Adenoviral vector	LT ADV-TK ganciclovir	Intravenous infusion	2	81	Completed No results reported to date	
5	NCT02561546	A Phase II Study to Investigate Preliminary Efficacy Using p53 Gene Therapy for Treatment of Diabetes Concurrent with Hepatocellular Carcinoma	This study aimed to investigate preliminary efficacy of p53 gene therapy in treatment of diabetes concurrent with HCC.	HCC	p53	Tumor suppressor	Recombinant adenoviral vector	p53 gene therapy TAE	Hepatic arterial injection following TAE	2	40	Not yet recruiting	
6	NCT00300521	Liver Transplantation With ADV-TK Gene Therapy Improves Survival in Patients with Advanced Hepatocellular Carcinoma	This study aimed to examine the effect of ADV-TK gene therapy combined with liver transplantation in patients with intermediate or advanced HCC.	HCC	HSVtk	Suicide	Adenoviral vector	ADV-TK	Intravenous infusion	2	40	Completed No results reported to date	
7	NCT00004178	Phase I Study of T Cells Modified with Chimeric AntiCEA Immunoglobulin-T Cell Receptors (IgTCR) in Adenocarcinoma	This study aimed to examine the effectiveness of ex vivo gene therapy in treating patients who have cancer that has not responded to previous therapy.	Primary Liver Cancers Metastatic Liver Tumors	CEA	Tumor-related Protein Coding	T Cells Modified with Chimeric Anti-CEA Immunoglobulin-T Cell Receptors (IgTCR) in Adenocarcinoma	Therapeutic autologous lymphocytes	Intravenous infusion	1		Completed No results reported to date	

8	NCT03313596	Multicenter Randomized Controlled Trial of Adenovirus-mediated Adjuvant Gene Therapy Improving Outcome of Liver Transplantation in Patients with Advanced Hepatocellular Carcinoma	This study aimed to compare the effect of liver transplantation plus suicide gene therapy versus liver transplantation only in advanced HCC.	HCC	HSVtk	Suicide	Adenoviral vector	ADV-TK<br>LT	Intravenous infusion	3	180	Recruiting No results available Estimated Completion Date: Dec, 2019	
9	NCT03480152	A Phase I/II Trial to Evaluate the Safety and Immunogenicity of a Messenger RNA (mRNA)-Based, Personalized Cancer Vaccine Against Neoantigens Expressed by the Autologous Cancer	This study aimed to examine the effect of mRNA-based, personalized cancer vaccine against neoantigens expressed by the autologous cancer.	Primary Liver Cancers	mRNA containing epitopes from immunogenic neoantigens	Tumor-related Protein Coding	mRNA vaccine	NCI-4650, a mRNA-based, Personalized Cancer Vaccine	Intramuscular injection	1/2	5	Terminated Related results partly reported	[159,160,161]
				Metastatic Liver Tumors	mRNA containing epitopes from immunogenic predicted neoantigens								
					mRNA containing epitopes from immunogenic mutations in tumor suppressor or driver genes								
10	NCT01967823	Phase II Study of Metastatic Cancer That Expresses NY-ESO-1 Using Lymphodepleting Conditioning Followed by Infusion of Anti-NY ESO-1 Murine TCR-Gene Engineered Lymphocytes	This study aimed to study the anti-tumor effect of T cell therapy by infusion of anti-NY-ESO-1 murine T-cell receptor-gene engineered lymphocytes.	HCC<br>Metastatic Liver Tumors	NY-ESO-1	Tumor-related Protein Coding	Anti-NY ESO-1 Murine TCR-Gene Engineered Lymphocytes	Anti-NY ESO-1 mTCR PBL Cyclophosphamide Fludarabine Aldesleukin	Intravenous infusion	2	43	Recruiting No results available Estimated Completion Date: July, 2028	[162,163,164]
11	NCT02509169	Trans-catheter Arterial p53-gene-embolization Using Gelatin Sponge Particles in Treatment of Patients with Advanced Hepatocellular Carcinoma: A Phase II Study	This study aimed to examine the effect of trans-catheter arterial embolization combined with p53 gene therapy in treatment of advanced HCC.	HCC	p53	Tumor suppressor	Recombinant adenoviral vector	TAE plus P53 gene TAE	Hepatic arterial injection following TAE	2	60	Recruiting No results available	
12	NCT02932956	Glypican 3-specific Chimeric Antigen Receptor Expressed in Autologous T Cells as Immunotherapy for Patients with Pediatric Solid Tumors	This study enrolls patients who have GPC3-positive liver cancers currently. Patients may be considered if the cancer has come back, has not gone away after standard treatment or the patient cannot receive standard treatment. This research study uses special immune system cells called GAP T cells, a new experimental treatment.	Pediatric Primary Liver Cancers	GPC-3	Tumor-related Protein Coding	CAR T cells	GAP T cells Cytoxan Fludara		1	18	Recruiting No results available Estimated Completion Date: Feb, 2037	
13	NCT00012155	A Phase I, Open-Label, Dose-Escalating Study of The Safety, Tolerability, And Anti-Tumor Activity of a Single Intrahepatic Arterial Injection of Genetically Engineered Herpes Simplex Virus, NV1020, In Subjects with Adenocarcinoma of The Colon with Metastasis to The Liver	This study aimed to examine the safety of oncolytic virus, NV1020, in patients with metastatic liver tumor of colon cancer, which showed no response to previous chemotherapy.	Metastatic Liver Tumors	Oncoytic Virus	Oncoytic Virus	oncolytic herpes simplex virus type-1(HSV-1)	NV1020, oncolytic herpes simplex virus type-1 (HSV-1)	Hepatic arterial injection	1		Completed Results partly reported	[74]
14	NCT00066404	A Phase I Clinical Trial of Intrapleural Adenoviral-Mediated Interferon-beta (IFN-ß) Gene Transfer for Pleural Malignancies	This study aimed to examine the effect of genetic immunotherapy using adenoviral vector delivering interferon-beta gene into a patient with metatstaic liver tumors.	Metastatic Liver Tumors	Interferon-beta	Genetic Immunotherapy	Adenoviral vector	recombinant adenovirus-hIFN-beta	Intrapleural injection	1		Active, not recruiting Results partly reported	[165]

15	NCT00035919	Tumor Site Specific Phase I Evaluation of Safety of Hepatic Arterial Infusion of a Matrix-Targeted Retroviral Vector Bearing a Dominant Negative Cyclin G1 Construct as Intervention for Colorectal Carcinoma Metastatic to Liver	This study aimed to examine the safety and anti-tumor effect of a gene therapy of dominant negative form of cyclin G1 for the metastatic liver tumors of colorectal cancer.	Metastatic Liver Tumors	Dominant Negative Cyclin G1	Tumor suppressor	Retroviral Vector	Mx-dnG1 Retroviral Vector	Hepatic arterial infusion	1/2	0	Withdrawn	
16	NCT00005629	Phase I/II Trial Testing Alpha Fetoprotein (AFP) Peptide Immunization in Hepatocellular Carcinoma	This study aimed to examine the anti-tumor effect and safety of gene vaccine therapy using AFP peptide in treating patients who have HCC or metastatic liver cancers.	Primary Liver Cancers Metastatic Liver Tumors	AFP	Tumor-related Protein Coding	AFP peptide	AFP gene hepatocellular carcinoma vaccine	Intradermal injection	1/2	6	Completed No results reported to date	
17	NCT02905188	Glypican 3-specific Chimeric Antigen Receptor Expressing T Cells as Immunotherapy for Patients with Hepatocellular Carcinoma	This study aimed to examine the effect of CAR-T cells expressing GPC-3 by ex vixo gene therapy.	HCC	GPC-3	Tumor-related Protein Coding	CAR T cells	GLYCAR T cells	Intravenous infusion	1	14	Recruiting No results available Estimated Completion Date: Oct, 2036	
18	NCT00093548	A Phase I/II Trial Testing Immunization with AFP + GM-CSF Plasmid Prime and AFP Adenoviral Vector Boost In Patients With Hepatocellular Carcinoma (AFP Prime-Boost Protocol)	This study aimed to examine the anti-tumor effect of gene vaccines using adenoviral vector expressing AFP and GM-CSF.	HCC	AFP, GM-CSF	Tumor-related Protein Coding	Adenoviral vector	Vaccination AFP plasmid DNA vaccine GM-CSF plasmid DNA hepatocellular carcinoma vaccine adjuvant	Intramuscular injection/Intradermal injection	1/2	0	Withdrawn	[92]
19	NCT01628640	Phase I Trial of Intratumoral Injection of Vesicular Stomatitis Virus Expressing Human Interferon Beta in Patients with Sorafenib Refractory/Intolerant Hepatocellular Carcinoma, Advanced Solid Tumors with Liver Predominant Locally Advanced/Metastatic Disease or Subcutaneous/Cutaneous Lesions	This study aimed to examine the effect of genetic immunotherapy using viral vector expressing interferon-beta gene.	Primary Liver Cancers Metastatic Liver Tumors	Interferon-beta	Genetic Immunotherapy	Vesicular Stomatitis Virus	Recombinant Vesicular Stomatitis Virus-expressing Interferon-beta	Intratumoral Injection	1	17	Active, not recruiting Estimated Completion Date: June, 2025	
20	NCT03602079	A Phase I-II, FIH Study of A166 in Locally Advanced/Metastatic Solid Tumors Expressing Human Epidermal Growth Factor Receptor 2 (HER2) or Are HER2 Amplified That Did Not Respond or Stopped Responding to Approved Therapies	This study aimed to examine the effect of antibody drug conjugate, A166, monotherapy in HER2-expressing or amplified patients who progressed on or did not respond to available standard therapies.	HCC CCC Metastatic Liver Tumors	HER-2	Tumor-related Protein Coding	Antibody Drug Conjugate (ADC)	A166, an Antibody Drug Conjugate (ADC) targeting HER2 expressing cancer cells.	Intravenous infusion	1/2	82	Recruiting No results available Estimated Completion Date: May, 2021	
21	NCT02416466	Phase Ib Trial of CAR-T Hepatic Artery Infusions Followed by Selective Internal Radiation Therapy (SIRT) With Yttrium-90 Sir-Spheres® for CEA-Expressing Liver Metastases	This study aimed to examine the effect of anti-CEA CAR-T cells hepatic artery infusions and yttrium-90 SIR-Spheres in patients with CEA-expressing liver metastases.	Metastatic Liver Tumors	CEA	Tumor-related Protein Coding	CAR-T cells	anti-CEA CAR-T cells	Hepatic arterial infusion	1	8	Completed No results reported to date	
22	NCT02869217	Phase Ib Study of TBI-1301 (NY-ESO-1 Specific TCR Gene Transduced Autologous T Lymphocytes) in Patients with Solid Tumors	This study aimed to examine the anti-tumor effect and safety of TBI-1301 (NY-ESO-1 Specific TCR Gene Transduced Autologous T Lymphocytes) in patients with solid tumors.	NY-ESO-1 Expressing Liver Cancers in HLA-A2 Positive Patients Metastatic Liver Tumors	NY-ESO-1	Tumor-related Protein Coding	NY-ESO-1 Specific TCR Gene Transduced Autologous T Lymphocytes	TBI-1301 (NY-ESO-1 Specific TCR Gene Transduced Autologous T Lymphocytes) Cyclophosphamide	Infusion	1	15	Recruiting No results available Estimated Completion Date: June, 2020	
23	NCT01061840	Phase I Trial of Bi-shRNAfurin and GMCSF Augmented Autologous Tumor Cell Vaccine for Advanced Cancer	This study aimed to examine the anti-tumor effect of autologous Vigil™ vaccine expressing granulocyte macrophage colony stimulating factor and bi-shRNAfurin from the Vigil™ plasmid.	Primary Liver Cancers Metastatic Liver Tumors	rhGMCSF and bi-shRNAfurin from the Vigil™ plasmid	Tumor-related Protein Coding	plasmid	Vaccination	Intradermal injection	1	100	Completed Results partly reported	[166,167]
24	NCT01437007	A Phase 1 Dose Escalation Study of Hepatic Intra-Arterial Administration of TKM 080301 (Lipid Nanoparticles	This study aimed to test the safety and effectiveness of TKM 080301 (lipid nanoparticles containing siRNA against the PLK1 gene	Metastatic Liver Tumors from Colorectal,	siRNA Against the PLK1	Oncogene suppression	Lipid Nanoparticles	TKM-080301	Hepatic arterial infusion	1	1	Completed Results partly reported	[53]

		Containing siRNA Against the PLK1 Gene Product) in Patients With Colorectal, Pancreas, Gastric, Breast, Ovarian and Esophageal Cancers With Hepatic	product) for cancer in the liver that has not responded to standard treatments.	Pancreas, Gastric, Breast, and Ovarian Cancers									
25	NCT03971747	A Phase 1 Study of AFP Specific T Cell Receptor Transduced T Cells Injection in Unresectable Hepatocellular Carcinoma	This study aimed to assess the safety and anti-tumor activity of C-TCR055 (AFP Specific T Cell Receptor Transduced T Cells Injection) injection in unresectable HCC patients.	HCC	AFP	Tumor-related Protein Coding	T Cell	AFP Specific T Cell Receptor T Cells	Intravenous infusion	1	9	Not yet recruiting	
26	NCT02418988	Multicenter, Open-labeled, Controlled Phase II Study: Trans-catheter Chemo-embolization Combined With rAd-p53 Gene Injection in Treatment of Advanced Hepatocellular Carcinoma	This study aimed to investigate the efficacy and safety usingTAE plus recombinant adenoviral human p53 gene (rAd-p53) in treatment of advanced HCC.	HCC	p53	Tumor suppressor	Recombinant adenoviral vector	TACE plus rAd-p53 artery injection TACE	Injected into the embolization artery.	2	120	Recruiting No results available	
27	NCT02850536	Phase Ib Trial of CAR-T Hepatic Artery Infusions or Pancreatic Venous Infusions Delivered with the Surefire Infusion System (SIS) for CEA-Expressing Liver Metastases or Pancreas Cancer	This study aimed to examine the safety and anti-tumor effect of anti-CEA CAR-T cell infusions delivered via the hepatic artery or splenic vein using the Surefire Infusion System (SIS) for patients with CEA-expressing liver metastases or pancreas cancer.	Metastatic Liver Tumors from Colorectal, Pancreas, Gastric, Breast, and Ovarian Cancers	CEA	Tumor-related Protein Coding	CAR-T cells	anti-CEA CAR-T cells	Hepatic arterial infusion or splenic vein	1	5	Active, not recruiting Estimated Completion Date: Dec, 2019	
28	NCT01373047	Phase I Trial of Intrahepatic Infusion Of 2nd Generation Designer T Cells for Cea-Expressing Liver Metastases	The study aimed to collect data on the safety and potential effectiveness of 2nd generation designer T cells delivered into the hepatic circulation in patients with liver metastases expressing the CEA tumor marker.	Metastatic Liver Tumors from Colorectal, Pancreas, Gastric, Breast, and Ovarian Cancers	CEA	Tumor-related Protein Coding	T cell	anti-CEA 2nd generation designer T cells	Hepatic arterial infusion or splenic vein	1	8	Completed No results reported to date	
29	NCT02432963	A Phase I Study of a p53MVA Vaccine in Combination with Pembrolizumab	This study aimed to examine the anti-tumor effect of genetic vaccines made from a gene-modified virus combined with pembrolizumab.	Adult Solid Neoplasm	p53	Tumor suppressor	Modified vaccinia virus	Vaccination		1	19	Active, not recruiting Estimated Completion Date: Feb, 2020	
30	NCT02715362	An Open-label, Uncontrolled, Single-arm Pilot Study to Evaluate Vascular Interventional Therapy Mediated GPC3-targeted Chimeric Antigen Receptor T Cells in Advanced Hepatocellular Carcinoma	This study aimed to examine the effect of CAR-T cells in the treatment of solid tumors by transcatheter arterial infusion, which is one kind of tumor intervention therapy pathway.	HCC	GPC3	Tumor-related Protein Coding	CAR-T cells	TAI-GPC3-CART cells	Hepatic arterial infusion	1/2	30	Recruiting No results available	
31	NCT00301106	Phase I Trial of Adenoviral Vector Delivery of the Human Interleukin-12 cDNA by Intratumoral Injection in Patients with Metastatic Breast Cancer to the Liver	This study aimed to examine the side effects and best dose of a gene-modified virus that can make interleukin-12 in treating women with metastatic liver tumors from breast cancer.	Metastatic Liver Tumors from Colorectal, Pancreas, Gastric, Breast, and Ovarian Cancers	Interleukin-12	Genetic Immunotherapy	Adenoviral Vector	adenovirus-mediated human interleukin-12	Intratumoral Injection	1	2	Terminated No results available	
32	NCT00072098	Phase I Trial of Adenoviral Vector Delivery of The Human Interleukin-12 cDNA By Intratumoral Injection in Patients with Metastatic Colorectal Cancer to The Liver	This study aimed to examine the safety and anti-tumor effect of interleukin-12 gene when injected into the tumors of patients with liver metastases from colorectal cancer.	Metastatic Liver Tumors from Colorectal, Pancreas, Gastric, Breast, and Ovarian Cancers	Interleukin-12	Genetic Immunotherapy	Adenoviral Vector	adenoviral vector-delivered interleukin-12	Intratumoral Injection	1	22	Terminated No results available	
33	NCT03198546	CAR-T Cell Targeting GPC3 for Immunotherapy of Hepatocellular Carcinoma: Phase I Clinical Trial	The study aimed to examine the safety, tolerance, and preliminary efficacy of the GPC3-T2-CAR-T cell immunotherapy in human HCC patients with GPC3 expression.	HCC	GPC3	Tumor-related Protein Coding	CAR-T cells	GPC3 targeting CAR-T cells	Systemic or local injections	1	30	Recruiting No results available Estimated Completion Date: Aug, 2022	[87]

34	NCT00103142	A Phase II Study of Active Immunotherapy with PANVAC or Autologous, Cultured Dendritic Cells Infected with PANVAC After Complete Resection of Hepatic or Pulmonary Metastases of Colorectal Carcinoma	This study aimed to examine the effect of gene vaccine therapy together with dendritic cells, how well it works compared to giving vaccine therapy together with GM-CSF in treating patients with liver or lung metastases from colorectal cancer removed by surgery.	Metastatic Liver Tumors from Colorectal, Pancreas, Gastric, Breast, and Ovarian Cancers	Vaccinia-Carcinoembryonic antigen (CEA)-mucin 1 (MUC-1)- Triad of costimulatory molecules TRICOM vaccine (PANVAC-V)	Tumor-related Protein Coding	Autologous dendritic cells	Vaccination		2	74	Completed Results available	[95]

CC, hepatocellular carcinoma; CCC, cholangiocellular carcinoma; TACE, transarterial chemoembolization; TAE, transarterial embolization; SVtk, thymidine kinase of herpes simplex virus; NY -ESO-1, New York esophageal squamous cell carcinoma 1; GPC-3, Glypican-3; LT, liver transplantation
